# Mr. Gladstone and the Prince of Wales

**Published:** 1889-05-11

**Authors:** 


### Mr. Gladstone and the Prince of Wales.

Mr. Gladstone, having read Mr. Burdett's public life of the Prince and Princess of Wales, entitled "Prince, Princess and People" (Longmans, Green and Co.), has written to thank him for it. Writing on the 29th of April, Mr. Gladstone says: "I have again to thank you for your kindness, and am glad on every ground that the public will now have an opportunity, through your work, of estimating the labours involved in the public life of the Prince and Princess of Wales." In a subsequent letter, dated the 1st of May, Mr. Gladstone writes: "The words I used were not intended for publication; but I have no objection to their being published if it is thought that this will be useful in making known the public life of their Royal Highnesses." The book may now be obtained from all the libraries throughout the country.

